# Downregulation of amplified in breast cancer 1 contributes to the anti-tumor effects of sorafenib on human hepatocellular carcinoma

**DOI:** 10.18632/oncotarget.8812

**Published:** 2016-04-18

**Authors:** Ming Li, Wei Wang, Yuzhen Dan, Zhangwei Tong, Wenbo Chen, Liping Qin, Kun Liu, Wengang Li, Pingli Mo, Chundong Yu

**Affiliations:** ^1^ State Key Laboratory of Cellular Stress Biology, Innovation Center for Cell Signaling Network, School of Life Sciences, Xiamen University, Xiamen, China; ^2^ Xiamen City Key Laboratory of Biliary Tract Diseases, Chenggong Hospital of Xiamen University, Xiamen, China; ^3^ Engineering Research Center of Molecular Diagnostics, Ministry of Education, School of Life Sciences, Xiamen University, Xiamen, China; ^4^ Department of Hepatobiliary Pancreas and Vessel Surgery, Chenggong Hospital of Xiamen University, Xiamen, China; ^5^ Department of Pathology, Chenggong Hospital of Xiamen University, Xiamen, China

**Keywords:** sorafenib, AIB1, HCC, cell death, mRNA translation

## Abstract

Multi-kinase inhibitor sorafenib represents a major breakthrough in the therapy of advanced hepatocellular carcinoma (HCC). Amplified in breast cancer 1 (AIB1) is frequently overexpressed in human HCC tissues and promotes HCC progression. In this study, we investigated the effects of sorafenib on AIB1 expression and the role of AIB1 in anti-tumor effects of sorafenib. We found that sorafenib downregulated AIB1 protein expression by inhibiting AIB1 mRNA translation through simultaneously blocking eIF4E and mTOR/p70S6K/RP-S6 signaling. Knockdown of AIB1 significantly promoted sorafenib-induced cell death, whereas overexpression of AIB1 substantially diminished sorafenib-induced cell death. Downregulation of AIB1 contributed to sorafenib-induced cell death at least in part through upregulating the levels of reactive oxygen species in HCC cells. In addition, resistance to sorafenib-induced downregulation of AIB1 protein contributes to the acquired resistance of HCC cells to sorafenib-induced cell death. Collectively, our study implicates that AIB1 is a molecular target of sorafenib and downregulation of AIB1 contributes to the anti-tumor effects of sorafenib.

## INTRODUCTION

Hepatocellular carcinoma (HCC) stands for one of the most challenging malignancies. HCC is currently the fifth most common cancer and the second leading cause of cancer-related death worldwide [1]. It is imperative to develop effective molecularly targeted agents to combat HCC.

Sorafenib is an oral multi-kinase inhibitor and the first Food and Drug Administration-approved molecularly targeted agent for patients with advanced HCC [2]. Despite sorafenib represents a major breakthrough in the therapy of advanced HCC, sorafenib treatment only increases 2-3 months longer overall survival compared with placebo treatment for advanced HCC patients and sorafenib treatment has a low response rate due to drug resistance or other reasons [3]. Therefore, clarification of the downstream regulators for the anti-tumor effects of sorafenib should be beneficial for the rational design of combination and individual therapy for HCC.

Sorafenib primarily targets and inhibits the growth factor receptor VEGFR and PDGFR as well as serine-threonine kinase Raf to suppress tumor proliferation and angiogenesis [4–6]. In addition to inhibition of tumor proliferation and angiogenesis, sorafenib can also induce tumor cell death to exert anti-tumor effects on various cancers. For instance, sorafenib can induce caspase-independent apoptosis in melanoma cells [7]. Sorafenib activates PUMA via GSK-3β and NF-κB pathway to promote cell apoptosis in colorectal cancer cells [8]. Sorafenib induces human leukemia cell apoptosis through down-regulation of Mcl-1 [9]. Consistently, sorafenib can cause HCC cell apoptosis through several molecular mechanisms, including activation of PUMA and BAD, induction of GADD45β as well as downregulation of c-IAP [10–13].

Amplified in breast cancer 1 (AIB1, SRC-3, RAC3, TRAM-1, ACTR and P/CIP) is a member of p160 coactivator family [14]. AIB1 not only interacts with nuclear hormone receptors but also interacts with other transcription factors to regulate the expression of their target genes. We previously found that AIB1 protein was frequently overexpressed in human HCC tissues and promoted HCC progression by enhancing cell proliferation and invasiveness [15]. Meanwhile, we also found that AIB1 could inhibit apoptosis and enhance chemoresistance in human cholangiocarcinoma cells [16]. Given that AIB1 plays an important role in HCC progression and chemoresistance, we investigate whether AIB1 is a downstream target of sorafenib for its anti-tumor effects. In the present study, we showed that AIB1 is a downstream target of sorafenib and AIB1 downregulation contributes to the anti-tumor effects of sorafenib.

## RESULTS

### Sorafenib downregulates AIB1 protein expression in HCC cells by inhibiting AIB1 mRNA translation

**Figure 1:**
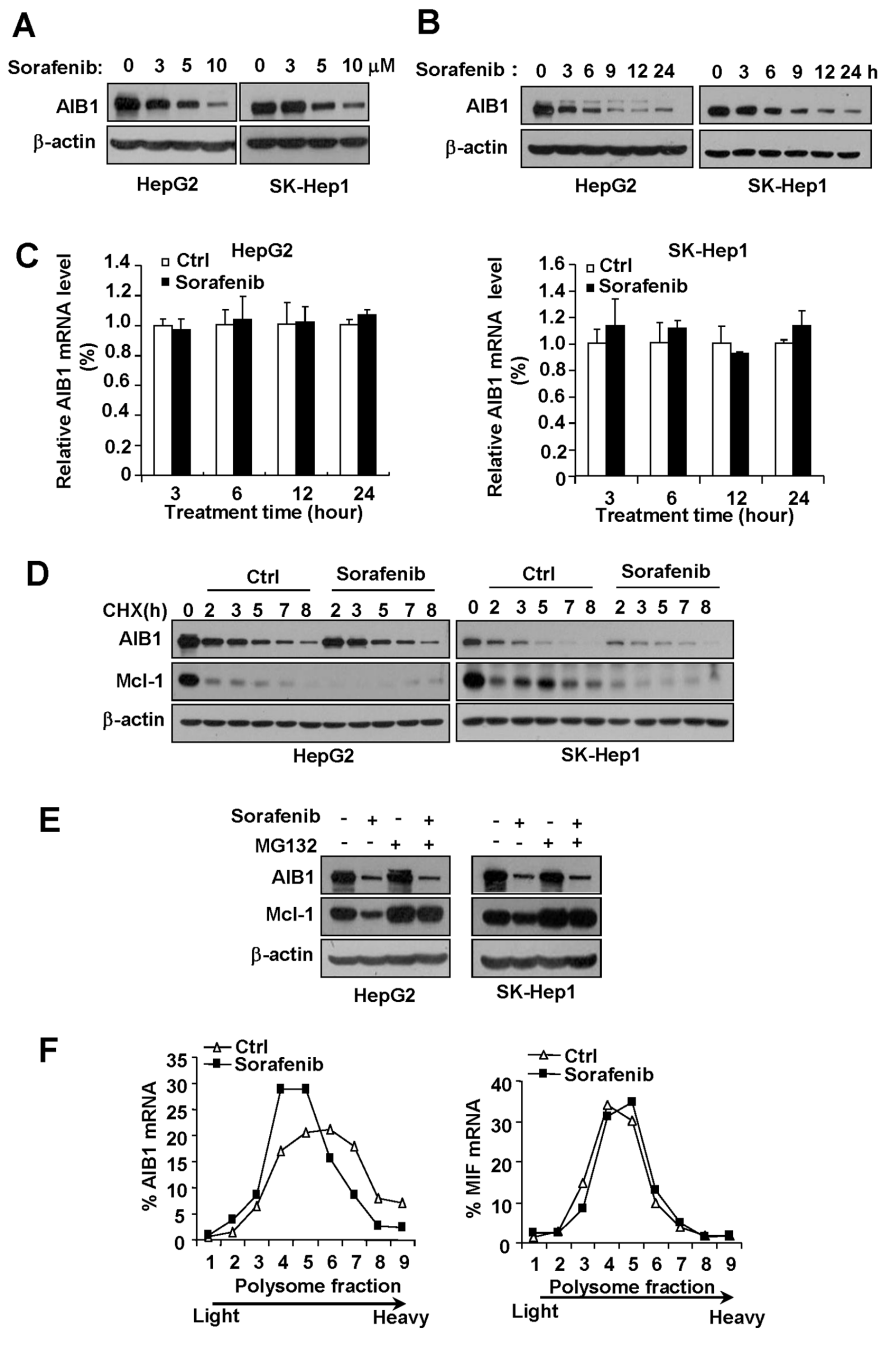
Sorafenib downregulates AIB1 protein expression in HCC cells by inhibiting AIB1 mRNA translation **A.** The levels of AIB1 protein were decreased after different concentrations of sorafenib treatment for 24 hours in HepG2 and Sk-Hep1 cells. **B.** The levels of AIB1 protein were downregulated by 10 μM sorafenib treatment for indicated time in HepG2 and Sk-Hep1 cells. **C.** The levels of AIB1 mRNA did not change after 5 μM sorafenib treatment for indicated time. All data are the mean +SD (n=3). **D.** Stability of AIB1 protein was not affected by 10 μM sorafenib treatment. **E.** sorafenib could still downregulate AIB1 protein expression in the presence of proteasome inhibitor MG132. **F.** Sorafenib inhibited AIB1 protein synthesis as demonstrated by polysomal RNA profile analysis.

To investigate the effects of sorafenib on AIB1 expression, HCC cell lines HepG2 and Sk-Hep1 were treated with different concentrations of sorafenib for 24 hours, and then cells were harvested for Western blot analysis. As shown in Figure 1A, the levels of AIB1 protein were significantly decreased in sorafenib-treated cells in a dose dependent manner, as compared to control cells. To examine the kinetics of sorafenib-mediated AIB1 downregulation, HepG2 and SK-Hep1 were treated with 10 μM sorafenib for different time. AIB1 downregulation was observed as early as 3 hours and reached a maximum at 24 hours during 24-hour sorafenib treatment (Figure 1B). These results suggest that sorafenib downregulates AIB1 protein expression in a dose and time dependent manner.

To examine whether AIB1 is downregulated at the transcriptional level, real-time PCR was performed to detect the mRNA levels of AIB1 in HepG2 and SK-Hep1 cells after sorafenib treatment. As shown in Figure 1C, the levels of AIB1 mRNA were not decreased by sorafenib treatment for different time even when AIB1 protein levels were significantly downregulated (24 hours) (Figure 1C). These results indicate that sorafenib downregulates AIB1 expression at the post-transcriptional level.

Given that AIB1 protein has a relative short half-life and can be degraded by ubiquitin-proteasome pathway [17], and sorafenib can affect the stability of proteins such as Mcl-1 through ubiquitin-proteasome pathway [18], we determined whether sorafenib affects the stability of AIB1 protein. Our results showed that sorafenib did not promote AIB1 protein degradation when cycloheximide (CHX) blocked protein synthesis, although it accelerated the degradation of Mcl-1 protein as expected (Figure 1D). In the presence of proteasome inhibitor MG132, sorafenib could still downregulate AIB1 protein expression, whereas it failed to downregulate Mcl-1 protein expression (Figure 1E). These results indicate that sorafenib does not decrease AIB1 protein stability and sorafenib downregulates AIB1 protein expression independent of ubiquitin-proteasome degradation pathway.

Since neither AIB1 mRNA levels nor AIB1 protein stability was affected by sorafenib, we speculated that downregulation of AIB1 protein by sorafenib was most likely due to a reduced mRNA-protein translation. To test this hypothesis, polysomal profile analysis of the translational status of AIB1 mRNAs was performed. Cytoplasmic extracts of control and sorafenib-treated HepG2 cells were fractionated over 20-50% sucrose gradients and evaluated for differences in distribution of the mRNAs of AIB1 and MIF by real-time PCR, respectively (Figure 1F). Sorafenib significantly decreased the amount of polysome-associated AIB1 mRNA as demonstrated by shifting AIB1 mRNA from high-density sucrose fractions (fractions 6-9) to low-density sucrose fractions (fractions 4 and 5) (Figure 1F, left panel), but did not affect the distribution of MIF (macrophage migration inhibitory factor) mRNA (Figure 1F, right panel), which has much shorter 5′ UTR and 3′ UTR for poor regulation of translation and serves as a reference. These results indicate that sorafenib downregulates AIB1 protein expression by inhibiting AIB1 mRNA translation.

### Downregulation of phosphorylated eIF4E and mTOR signaling contributes to sorafenib-induced inhibition of AIB1 translation

**Figure 2:**
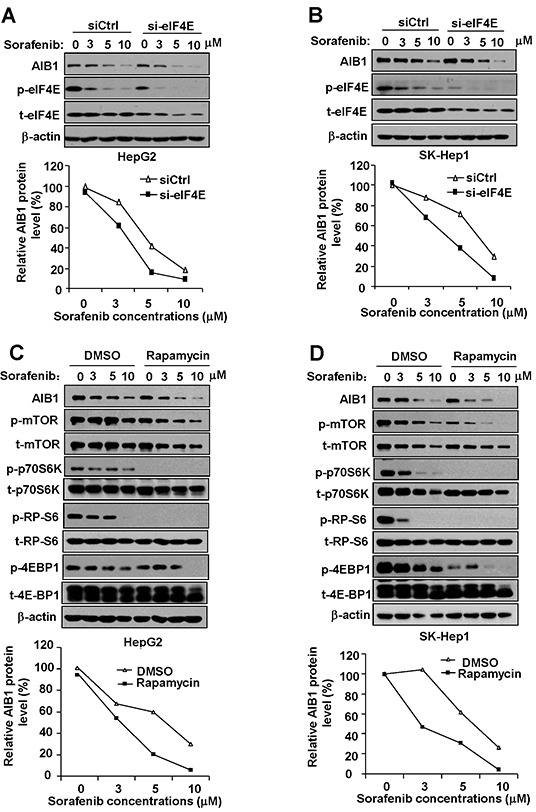
Downregulation of phosphorylated eIF4E and mTOR signaling contributes to sorafenib-induced inhibition of AIB1 translation **A.** and **B.** Downregulation of eIF4E enhanced sorafenib-induced inhibition of protein expression of AIB1 and phospho-eIF4E in HCC cells. **C.** and **D.** mTOR signaling pathway inhibitor rapamycin enhanced sorafenib-induced downregulation of AIB1 protein.

The mRNA-protein translation is the most expensive process and is tightly controlled at the level of initiation in a cell [19]. Recruitment of eukaryotic initiation factors 4e (eIF4E) for formation of eIF4F complex is the major point of regulation at the level of translation [20]. Upon recruitment, eIF4E is phosphorylated by the kinase MNK, which is activated by ERKs. Phosphorylation of eIF4E enhances its affinity for mRNA cap and plays a key regulatory role in translation initiation [20, 21]. Our RNA immunoprecipitation assay result showed that AIB1 mRNA could interact with eIF4E as expected (Supplementary Figure 1). To determine whether the inhibition of AIB1 mRNA translation by sorafenib is due to the inhibition of eIF4E phosphorylation, we examined the protein levels of total and phosphorylated eIF4E (phospho-eIF4E) in HepG2 and SK-Hep1 cells after sorafenib treatment. As shown in Figure 2A and 2B, sorafenib significantly suppressed phospho-eIF4E protein expression in a dose dependent manner, whereas it had less effect on the total eIF4E protein levels. Therefore, sorafenib may suppress AIB1 translation through inhibiting phosphorylation of eIF4E. Furthermore, we used eIF4E siRNA to knock down eIF4E expression and then examined the protein levels of phospho-eIF4E and AIB1 after sorafenib treatment. Knockdown of eIF4E significantly enhanced sorafenib-induced downregulation of phospho-eIF4E and AIB1 protein expression (Figure 2A and 2B). These results suggest that downregulation of phosphorylated eIF4E contributes to sorafenib-induced inhibition of AIB1 translation.

As a central serine/threonine protein kinase, mTOR controls protein synthesis via the phosphorylation of two downstream effectors: the p70S6K which activates RP-S6, and the 4E-BP1 which is inactivated by phosphorylation to induce the release of eIF4E [22]. It is reported that sorafenib can inhibit mTOR signaling [23]. We therefore tested whether mTOR signaling is inhibited by sorafenib and involved in sorafenib-induced downregulation of AIB1 protein expression. In parallel with the downregulation of AIB1 protein, sorafenib inhibited the expression of phospho-p70S6K, phospho-RP-S6, and phospho-4E-BP1 in HCC cells (Figure 2C and 2D), suggesting that sorafenib indeed can inhibit mTOR/p70S6K/RP-S6/4E-BP1signaling. To test whether mTOR/p70S6K/RP-S6/4E-BP1signaling is involved in the regulation of AIB1 protein expression, we treated HCC cells with sorafenib and mTOR inhibitor rapamycin. Rapamycin treatment significantly downregulated the expression of phospho-p70S6K, phospho-RP-S6 and phospho-4E-BP1 and enhanced sorafenib-induced AIB1 protein expression (Figure 2C and 2D). These results suggest that downregulation of mTOR/p70S6K/RP-S6/4E-BP1signaling also contributes to sorafenib-induced inhibition of AIB1 translation.

### Simultaneous downregulation of phosphorylated eIF4E and mTOR/p70S6K/RP-S6/4E-BP1 signaling is required for downregulation of AIB1 protein expression

**Figure 3:**
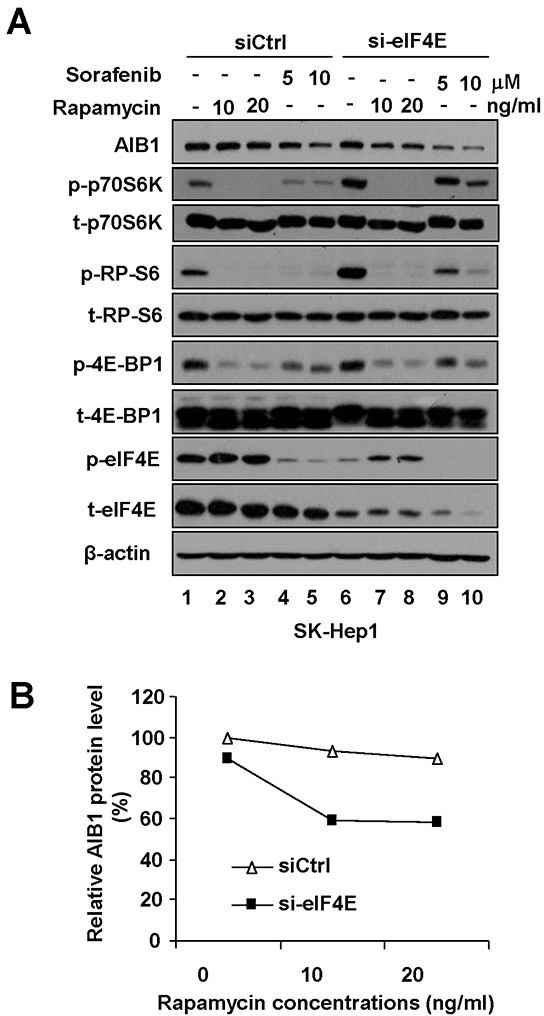
Simultaneous downregulation of phosphorylated eIF4E and mTOR/p70S6K/RP-S6/4E-BP1 signaling is required for downregulation of AIB1 protein expression **A.** After SK-Hep1 cells were transfected with control siRNA or eIF4E-specific siRNA for 48 hours, Cells were treated with rapamycin or sorafenib for 24 hours and related protein levels were detected by Western blot analysis. **B.** Quantitative analysis of relative AIB1 protein expression after eIF4E-specific siRNA and/or rapamycin treatment.

Although either knockdown of eIF4E or rapamycin treatment could significantly enhance sorafenib-induced downregulation of AIB1 protein expression, knockdown of eIF4E alone or rapamycin treatment alone could not significantly downregulate AIB1 protein expression (Figure 2A–2D, compare lane 1 with lane 5). To explore the underlying mechanisms, we treated SK-Hep1 cells with eIF4E-specific siRNA, rapamycin, and eIF4E-specific siRNA plus rapamycin, respectively, and then examined the protein levels of AIB1, phospho-eIF4E, phospho-p70S6K, phospho-RP-S6, and phospho-4E-BP1. We found that although rapamycin alone effectively suppressed the expression of phospho-p70S6K, phospho-RP-S6 and phospho-4E-BP1, (Figure 3A, compare lanes 2 and 3 with lane 1), it boosted the expression of phospho-eIF4E (Figure 3A compare lanes 2 and 3 with lane 1 or compare lanes 7 and 8 with lane 6), which was partially in line with previous report [24]. Therefore, we speculated the reason rapamycin alone does not inhibit AIB1 protein expression is that upregulation of phospho-eIF4E may counteract the suppressed effect of rapamycin on AIB1 protein expression. Indeed, when eIF4E-specific siRNA efficiently reduced the expression of phospho-eIF4E (Figure 3A, compare lanes 7 and 8 with lanes 2 and 3), rapamycin could inhibit AIB1 protein expression (Figure 3A, compare lanes 6, 7, 8 with lanes 1, 2, 3, and Figure 3B). Furthermore, we found although eIF4E siRNA alone effectively reduced the expression of phosphorylated eIF4E, it significantly upregulated phospho-p70S6K and phospho-RP-S6 (Figure 3A, compare lane 6 with lane 1). This may explain why downregulation of phospho-eIF4E alone did not lead to the downregulation of AIB1 protein (Figure 3A, compare lane 6 with lane 1), since upregulation of phospho-p70S6K and phospho-RP-S6 may counteract its downregulation effect on AIB1 protein expression. Collectively, our data suggest that simultaneous downregulation of phosphorylated eIF4E and mTOR/p70S6K/RP-S6/4E-BP1 signaling is required for downregulation of AIB1 protein expression, which can be achieved by eIF4E-specific siRNA plus rapamycin treatment or sorafenib treatment alone (Figure 3A).

### Downregulation of AIB1 contributes to sorafenib-induced HCC cell death

**Figure 4:**
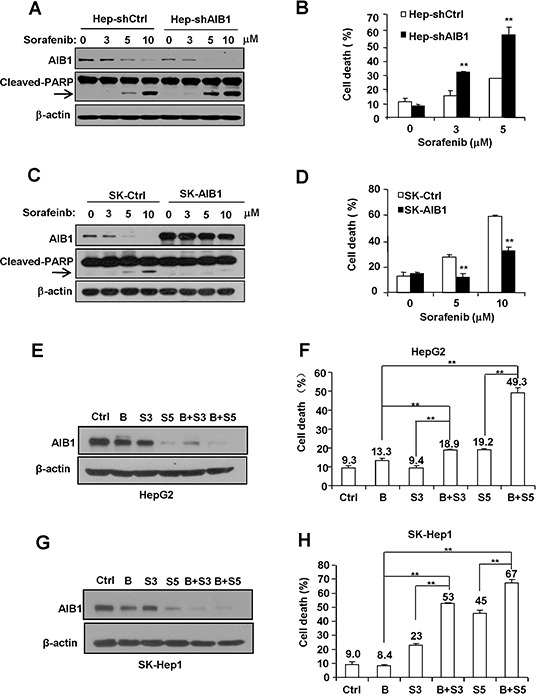
Downregulation of AIB1 contributes to sorafenib-induced HCC cell death **A.** and **B.** Downregulation of AIB1 increased sorafenib-induced cell death. (A) The protein levels of AIB1 and apoptotic mark protein cleaved-PARP were detected by Western blot analysis. (B) Cell death was evaluated by flow cytometry. **C.** and **D.** Overexpression of AIB1 decreased sorafenib-induced cell death. (C) The protein levels of AIB1 and cleaved-PARP were detected by Western blot analysis. (D) Cell death was evaluated by flow cytometry. **E.** AIB1 inhibitor bufalin (10 nM) enhanced sorafenib-induced downregulation of AIB1 protein in HepG2 cells. **F.** Bufalin enhanced sorafenib-induced cell death in HepG2 cells. **G.** Bufalin enhanced sorafenib-induced downregulation of AIB1 protein in SK-Hep1 cells. **F.** Bufalin enhanced sorafenib-induced cell death in SK-Hep1 cells. B stands for bufalin (10 nM), S stands for sorafenib, S3 stands for 3 μM sorafenib, S5 stands for 5 μM sorafenib; All data are the mean + SD (n=3). **p < 0.01.

To determine the effects of AIB1 on sorafenib-induced HCC cell death, HepG2 cells were stably transfected with pSUPER vector (shCtrl) or AIB1-knockdown vector pSUPER-shAIB1 (shAIB1), whereas SK-Hep1 cells which express relatively less AIB1 protein were stably transfected with pCR3.1 vector (Ctrl) or AIB1-expression vector pCR3.1-AIB1 (AIB1), and then these cells were treated with sorafenib for 24 hours. As shown in Figure 4A and 4B, stable knockdown of AIB1 in HepG2 cells significantly increased sorafenib-induced cell death as demonstrated by increased cleaved PAPR (poly ADP-ribose polymerase) expression and increased dead cells. In contrast, stable overexpression of AIB1 in SK-Hep1 cells significantly decreased sorafenib-induced cell death as demonstrated by reduced cleaved PAPR (poly ADP-ribose polymerase) expression and reduced dead cells (Figure 4C and 4D). These results implicate that sorafenib-induced downregulation of AIB1 contributes to sorafenib-induced cell death.

Recently, Wang *et al.* reported that the bufalin is a potent small molecule AIB1 inhibitor that can strongly decrease the protein levels of AIB1 and inhibit cancer cell proliferation [25]. To examine whether bufalin could enhance sorafenib-induced AIB1 downregulation and cell death, HepG2 and SK-Hep1 cells were treated with bufalin, sorafenib, and bufalin plus sorafenib for 24 hours, respectively. As shown in Figure 4E–4H, bufalin alone could downregulate AIB1 protein levels as expected; and bufalin could enhance sorafenib-induced AIB1 downregulation and cell death. These results implicate that combination of AIB1 inhibitors and sorafenib has additive or synergistic anti-tumor effects on HCC.

### Downregulation of AIB1 contributes to sorafenib-induced cell death through increasing the levels of intracellular reactive oxygen species (ROS) in HCC cells

**Figure 5:**
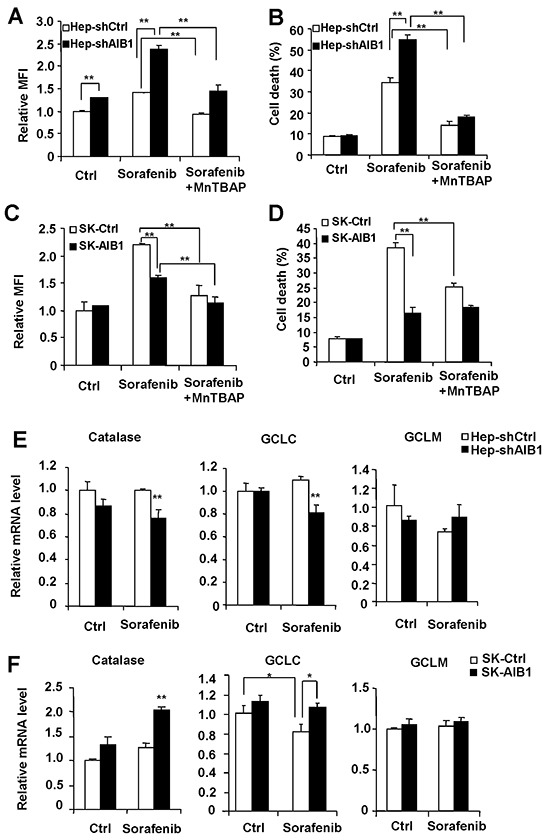
Downregulation of AIB1 contributes to sorafenib-induced cell death through increasing the levels of intracellular ROS in HCC cells **A.** Downregulation of AIB1 increased sorafenib-induced ROS in HepG2 cells. **B.** MntBAP decreased sorafenib-induced cell death in HepG2 cells. **C.** Overexpression of AIB1 decreased sorafenib-induced ROS in SK-Hep1 cells. **D.** MnTBAP decreased sorafenib-induced cell death in SK-Hep1 cells. **E.** Downregulation of AIB1 decreased the mRNA levels of catalase and GCLC after sorafenib treatment. **F.** Overexpression of AIB1 increased the mRNA levels of catalase and GCLC after sorafenib treatment. All data are the mean + SD (n=3). *p < 0.05,**p < 0.01.

Since sorafenib-induced cell death is partially dependent on sorafenib-induced ROS production in HepG2 cells [26], and AIB1 can inhibit intracellular ROS levels in human cholangiocarcinoma cells [16], we hypothesized that sorafenib-mediated downregulation of AIB1 contributes to sorafenib-induced intracellular ROS production and corresponding cell death in HCC cells. To test it, we investigated the effects of downregulation or upregulation of AIB1 on sorafenib-induced ROS levels and cell death in HepG2 and SK-Hep1 cells, respectively. The results showed that knockdown of AIB1 enhanced sorafenib-induced intracellular ROS and cell death in HepG2 cells (Figure 5A and 5B), whereas overexpression of AIB1 significantly decreased sorafenib-induced intracellular ROS levels and cell death in SK-Hep1 cells (Figure 5C and 5D). These data indicate that the levels of intracellular ROS are regulated by AIB1 and it might contribute to sorafenib-induced cell death in HCC cells. To further confirm that sorafenib-induced HCC cell death is due in part to increased ROS, HCC cells were treated with sorafenib in the absence or presence of antioxidant MnTBAP, and then ROS levels and cell death were evaluated by flow cytometry. As shown in Figure 5A and 5C, MnTBAP efficiently decreased sorafenib-induced ROS levels in both HepG2 and SK-Hep1 cells. Meanwhile, MnTBAP significantly blocked sorafenib-induced cell death in both HepG2 and SK-Hep1 cells, and abolished the effects of AIB1 on cell death (Figure 5B and 5D). These results indicate that increased intracellular ROS is indeed responsible for sorafenib-induced cell death.

To determine the mechanisms by which AIB1 affects intracellular ROS levels, we detected the mRNA levels of some enzymes that can regulate intracellular ROS balance, including the catalase that decreases endogenous hydrogen peroxide, the catalytic subunit of glutamate cysteine ligase (GCLC) and the modifier subunit of glutamate cysteine ligase (GCLM) that promote intracellular ROS scavenge. As shown in Figure 5E, AIB1-knockdown HepG2 cells had reduced levels of catalase and GCLC compared to control cells after sorafenib treatment. Conversely, AIB1-overespressed SK-Hep1 cells had higher levels of catalase and GCLC than control cells after sorafenib treatment (Figure 5F). These results suggest that the expression of catalase and GCLC in the presence of sorafenib is regulated by AIB1, and downregulation of AIB1 by sorafenib may at least in part be responsible for sorafenib-induced ROS.

### Resistance to sorafenib-mediated downregulation of AIB1 contributes to the acquired resistance of HCC cells to sorafeinb-induced cell death

**Figure 6:**
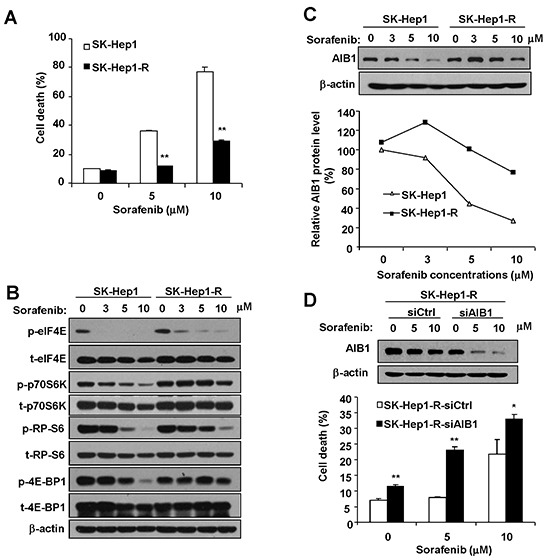
Resistance to sorafenib-mediated downregulation of AIB1 protein contributes to the acquired resistance of HCC cells to sorafeinb-induced cell death **A.** Resistant cells (SK-Hep1-R) were resistant to sorafenib-induced cell death. **B.** SK-Hep1-R cells were less sensitive to sorafenib-induced inhibition of eIF4E and p70S6K/RP-S6/4EBP1 signaling. **C.** SK-Hep1-R cells were less sensitive to sorafenib-induced inhibition of AIB1 expression. **D.** Downregulation of AIB1 promoted sorfenib-induced cell death in SK-Hep1-R cells. All data are the mean + SD (n=3). *p < 0.05, **p <0.01.

Acquired resistance of HCC cells to sorafenib is one of the major problems that limits the effectiveness of sorafenib used to treat HCC. To investigate the molecular mechanisms of acquired resistance to sorafenib-induced cell death, we established sorafenib-resistant SK-Hep1 cell lines by exposing cells to sorafenib at low doses escalating to higher doses for a long period of time. We examined the cytotoxic effects of sorafenib using flow cytometric assay. As shown in Figure 6A, sorafenib-resistant SK-Hep1 (SK-Hep1-R) cells exhibited reduced cell death compared with wild-type SK-Hep1 cells after sorafenib treatment as measured by flow cytometric assay. Furthermore, we found that SK-Hep1-R cells showed a resistance to sorafenib-mediated downregulation of phosphorylation levels of eIF4E, p70S6K, RP-S6, and 4E-BP1, as compared to wild-type SK-Hep1 cells (Figure 6B), indicating that the acquired resistance of SK-Hep1-R cells to sorafenib-induced cell death might be due in part to the resistance to sorafenib-induced downregulation of eIF4E and p70S6K/RP-S6 signaling. Resistance to sorafenib-induced downregulation of eIF4E and p70S6K/RP-S6 signaling might lead to the resistance to sorafenib-induced downregulation of AIB1 in SK-Hep1-R cells. Indeed, as shown in Figure 6C, resistance to sorafenib-mediated downregulation of AIB1 was observed in SK-Hep1-R cells, as compared with SK-Hep1 cells. To determine whether resistance to sorafenib-mediated downregulation of AIB1 contributes to the acquired resistance of SK-Hep1-R cells to sorafenib, we knocked down AIB1 in SK-Hep1-R cells and then measured sorafenib-induced cell death. As shown in Figure 6D, knockdown of AIB1 restored the sensitivity of SK-Hep1-R cells to sorafenib-induced cell death to some extent, indicating that sorafenib-induced downregulation of AIB1 protein contributes to the acquired resistance of HCC cells to sorafenib-induced cell death.

### Downregulation of AIB1 enhances the anti-tumor effects of sorafenib *in vivo*

**Figure 7:**
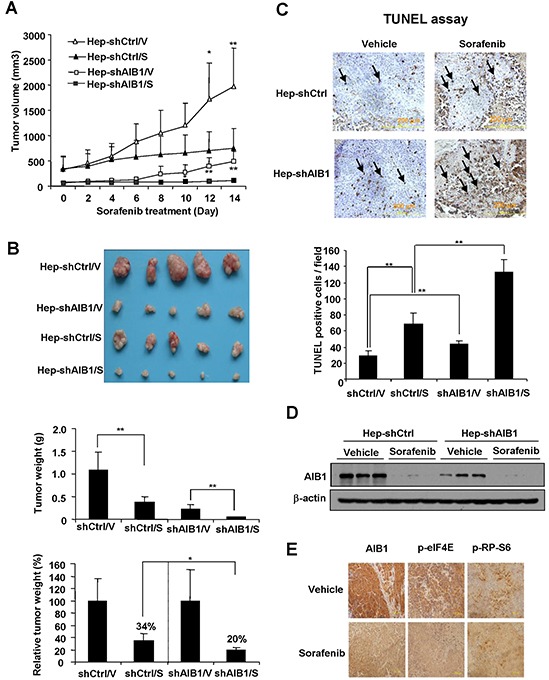
Downregulation of AIB1 enhances the anti-tumor effects of sorafenib *in vivo* **A.** Both AIB1 knockdown and sorafenib treatment inhibited HCC tumor growth. n=5; *p < 0.05; **p < 0.01. **B.** AIB1 knockdown enhanced the inhibitory effects of sorafenib on tumor growth. n=5; *p<0.05; **p<0.01 **C.** AIB1 knockdown enhanced sorafenib-induced xenograft tumor cell apoptosis. Tumor cell apoptosis was detected by TUNEL staining. n=4; **p<0.01. **D.** Sorafenib downregulated AIB1 protein expression in xenograft tumors. **E.** Immunohistochemical analysis of AIB1, p-eIF4E and p-RP-S6 protein expression in representative tumors treated with vehicle or sorafenib.

To determine whether downregulation of AIB1 contributes to the anti-tumor effects of sorafenib *in vivo*, AIB1-knockdown HepG2 cells (Hep-shAIB1) and control HepG2 cells (Hep-shCtrl) were injected subcutaneously into nude mice to establish xenograft tumors. Sixteen days later, mice were treated with 25 mg/kg/day of sorafenib or control vehicle by oral gavage for fourteen consecutive days. As shown in Figure 7A, Hep-shAIB1 tumors grew much slower than Hep-shCtrl tumors in the absence of sorafenib, demonstrating that down-regulation of AIB1 inhibits HCC growth, which is consistent with our previous report [15]. In the presence of sorafenib, the growth of both Hep-shCtrl and Hep-shAIB1 tumors was inhibited (Figure 7A). At the end of the study (day 14), while the tumor weight of sorafenib-treated Hep-shCtrl group (370 ± 130 mg) was 34% of the vehicle-treated Hep-shCtrl group (1100 ± 400 mg) (Figure 7B), the tumor weight of sorafenib-treated Hep-shAIB1 group (45 ± 10 mg) was only 20% of the vehicle-treated Hep-shAIB1 group (220 ±110 mg) (Figure 7B). Furthermore, TUNEL assay showed that downregulation of AIB1 not only increased basal cell apoptosis in tumor tissues but also significantly promoted sorafenib-induced cell apoptosis in tumor tissues (Figure 7C). Consistent with the *in vitro* findings, Western blot results showed that sorafenib significantly decreased the protein levels of AIB1 in both Hep-shCtrl and Hep-shAIB1 tumors (Figure 7D). Furthermore, immunohistochemical results showed that sorafenib inhibited AIB1, p-eIF4E and p-RP-S6 protein expression in tumors (Figure 7E). Collectively, these results demonstrate that downregulation of AIB1 enhances the anti-tumor effects of sorafenib *in vivo*.

## DISCUSSION

Our study reports for the first time that sorafenib could significantly downregulate AIB1 protein expression by inhibiting AIB1 mRNA translation in HCC cells. The eIF4E is a rate-limiting factor responsible for delivering cellular mRNA to the eIF4F complex to facilitate ribosomal loading and mRNA translation. The phosphorylation of eIF4E can promote its cap affinity and thereby enhance translation [20, 21, 27]. The mTOR signaling pathway also plays an important role in protein synthesis, it can activate 4E-BP1/eIF4E and p70S6K/RP-S6 signaling to promote protein translation [22]. In our study, mTOR inhibitor rapamycin or eIF4E-specific siRNA alone did not inhibit AIB1 protein expression, but eIF4E-specific siRNA plus rapamycin treatment can inhibit AIB1 protein expression. These results suggest that simultaneous downregulation of eIF4E and mTOR/p70S6K/RP-S6/4E-BP1 signaling by sorafenib is required for inhibition of AIB1 translation.

The eIF4E is involved in a wide spectrum of mRNA translation. When the molar concentration of mRNA exceeds eIF4E, mRNA has to compete for eIF4E to gain access to the translation machinery. Weak mRNAs (harboring long upstream AUGs, highly structured 5′-UTR; such as oncogenes and growth factors) are particularly dependent on excess eIF4E for efficient translation compared with strong mRNAs (harboring short, unstructured 5′-UTR; such as housekeeping genes) because of different efficiency of eIF4F scanning and recognition of start codon [20, 27, 28]. According to criterion of classification and regression tree (CART) [28], we found that AIB1 mRNA harbored a typical weak translated-5'UTR (low free energies; 3 upstream AUGs), therefore, the sensitivity of AIB1 to sorafenib-mediated translational inhibition may be due to its relative week mRNA.

Our previous study showed that AIB1 was frequently overexpressed in HCC tissues and played an important role in HCC progression [15]. Therefore, sorafenib-induced AIB1 downregulation may enhance the anti-tumor effects of sorafenib on HCC. Indeed, our results demonstrated that enforced expression of AIB1 significantly reduced cell death caused by sorafenib, whereas knockdown of AIB1 remarkably promoted sorafenib-induced cell death both *in vitro* and *in vivo*. These data indicate that sorafenib-induced AIB1 downregulation contributes to cell death. In addition to promoting cell death, sorafenib can execute its anti-tumor effects through inhibition of the proliferation and invasion of tumor cells [4–6]. Since AIB1 can promote the proliferation and invasion of HCC cells [15], the inhibitory effects of AIB1 downregulation on HCC cell proliferation and invasion may also contribute to the anti-tumor effects of sorafenib on HCC.

Acquired resistance of HCC cells to sorafenib is one of the major problems that limits the effectiveness of sorafenib used to treat HCC. Therefore, the molecular mechanisms of the acquired resistance of HCC cells to sorafenib were extensively investigated. A research in 23 HCC cell lines showed that the levels of phosphorylated RP-S6 were significantly correlated with the resistance of HCC cells to sorafenib [29]. Other reports showed that activation of PI3K/Akt and p38 MAPK signaling pathways mediates the acquired resistance to of HCC cells to sorafenib [30, 31]. Our results showed that the inhibition of p70S6K/RP-S6 signaling by sorafenib were less sensitive in resistant cells compared with wild-type cells after sorafenib treatment, which is in part consistent with those reports. Resistance to sorafenib-induced downregulation of eIF4E and p70S6K/RP-S6 signaling in our sorafenib-resistant cells led to the resistance to sorafenib-mediated downregulation of AIB1, which contributes to the acquired resistance of HCC cells to sorafeinb-induced cell death.

Taken together, our study demonstrates that AIB1 is a target of sorafenib and downregulation of AIB1 contributes to the anti-tumor effects of sorafenib on HCC. Our findings help to understand the anti-tumor mechanisms of sorafenib, and benefit for the rational design of personalized therapy or combination therapy for HCC by using sorafenib alone or sorafenib combined with other anti-tumor reagents.

## MATERIALS AND METHODS

### Cell culture

HCC cell lines HepG2 and SK-Hep1 were cultured in high glucose DMEM (HyClone) supplemented with 10% fetal bovine serum (HyClone) and 100 U/ml penicillin and 100 mg/ml streptomycin and were maintained in the humidified incubator with 95% air and 5% CO2 at 37°C.

### Reagents and antibodies

Sorafenib tosylate was purchased from LC Laboratories (Boston, MA) and was dissolved in DMSO. Cycloheximide (CHX) and MG132 were obtained from Sigma Aldrich and dissolved in ethanol and DMSO, respectively. Antioxidant MnTBAP (Mn(III) tetrakis (4-benzoic acid) porphyrin chloride) was purchased from Merck Millipore. Antibodies for AIB1 (5E11), eIF4E, phospho-eIF4E (Ser209), mTOR, phosphor-mTOR(Ser2448), p70S6K, phospho-p70S6K (Thr389), RP-S6, phospho-RP-S6 (Ser235/236), 4EBP1, phospho-4E-BP1 (Ser65) were purchased from Cell Signaling Technologies. Antibodies for PARP, Mcl-1 were from Santa Cruz Biotechnology. β-actin antibody was purchased from Sigma.

### Small interfering RNA and stable cell lines

eIF4E siRNA (sense: GGAUGGUAUUGAGCCUAUG, antisense: CAUAGGCUCAAUACCAUCC) [32] and nonspecific siRNA were purchased from Invitrogen. siRNAs were transfected with lipofectamine2000 to knock down eIF4E protein expression in HCC cell lines following the manufacturer's instruction. To establish stable AIB1-overexpression cell lines, SK-Hep1 cells were transfected with pCR3.1-FlAG-AIB1 and pCR3.1 control vector, respectively. Stably transfected cells were selected with 1mg/ml G418. To develop stable AIB1-knockdown cell lines, HepG2 cells were transfected with pSUPER-shAIB1and pSUPER control vector [15], respectively. Stably transfected cells were selected with 1 μg/ml puromycin.

### Cell death assay

The cell death assay was analyzed by propidium iodide (PI) staining. Briefly, the cells were harvested and collected in phosphate-buffered saline (PBS). After washing with PBS, the cells were resuspended in 1 ml PBS containing 5 μg PI. PI incorporation and cell size were quantified by flow cytometry. All cells were divided into three regions. PI-negative cells of normal size were considered viable cells; PI-positive and smaller size cells were considered apoptotic cells of early phase and PI-negative cells of smaller size were consider died cells of later period, and the last two regions were consider cell death.

### Apoptosis assay

The apoptosis of xenograft tumors was detected by TUNEL assay using ApopTag® Peroxidase *In Situ* Apoptosis Detection Kit from Millipore (S7100) according to the manufacturer's instruction.

### Reactive oxygen species (ROS) assay

ROS assay was performed as previously described [16]. Briefly, intracellular H_2_O_2_ was measured by 2′, 7′-dichlorofluorescein (DCF) fluorescence. The cells were incubated with 10 μM 2′, 7′-dichlorofluorescein diacetate (DCFH-DA, Sigma), which is taken up and oxidized to the fluorescent DCF by intracellular H_2_O_2_ at 37°C for 30 min and then the cells were immediately analyzed by flow cytometry.

### Western blot analysis and real-time RT-PCR

Western blot analysis and real-time PCR were performed as previously described [15]. The primers used for real-time PCR are listed in Supplementary Table 1.

### Polysomal RNA profile analysis

Polysomal RNA profile analysis was performed as previously described [33]. In brief, after sorafenib treatment, cell lysates were separated in a 20-50% sucrose gradient and nine fractions were collected per gradient. The absorbance of 260 nm was monitored in each fraction and RNA was isolated from each fraction. The mRNA levels of AIB1 and MIF in individual fraction from control and sorafenib- treated groups were determined by real-time PCR.

### Tumor xenograft experiments

The protocols for the *in vivo* studies were approved by Institutional Animal Care and Use Committee of Laboratory Animal Center of Xiamen University. 4-6 week old male nude mice were obtained from Laboratory Animal Center of Xiamen University. Nude mice were injected subcutaneously in both flanks with 2×10^6^ HepG2-shAIB1 cells or control cells, respectively. Sixteen days after cell injection, nude mice were orally administered with sorafenib tosylate for 14 days in a dose of 25 mg/kg/day. The volume of the tumor was monitored and calculated following the formula: Volume=length×Width2×0.52. Tumor were harvested and weighed, and then were dissected and fixed in 10% formalin and embedded in paraffin for TUNEL. Remaining tumors were homogenized in RIPA for Western blot analysis.

### Immunohistochemistry

Slides were soaked in preheated citrate buffer (pH 6.0) and heated in a microwave for 20 minutes to retrieve antigen. After cooling, slides were washed with PBS for three times, and then incubated with AIB1 antibody (1:100; Santa Cruz), p-eIF4E antibody (1:100; Santa Cruz) or p-RP-S6 antibody (1:100; Cell Signaling) for over night at 4°C. On the second day, slides were washed with PBS for three times, and then incubated with an horseradish peroxidase-conjugated secondary antibody for 1 h at room temperature. After washing, DAB reagent was added to visualize labeled protein.

### RNA Immunoprecipitation Assay

RNA immunoprecipitation assay was performed as described [34]. Briefly, HepG2 cells were washed with PBS and harvested in lysis buffer for 20 minutes. After centrifugation, the supernatant was precleared with normal immunoglobulin G (IgG) and 20 μl protein A/G agarose beads for 1.5 h. Protein A/G beads (20 μl) were incubated with 2 μg IgG or 2μg anti-eIF4E antibody (Cell Signaling) for 2 h at room temperature, and then the cell lysates were incubated with the above mix for 2 h at room temperature. Total RNAs binding to protein A/G beads were isolated and the mRNA levels of AIB1 were determined by real-time PCR.

### Statistical analysis

All data were shown as the mean + SD from the number of replicates described in results. The statistical significant effects between mean values (p <0.05) were assessed with the two-tailed Student's t-test in SPSS.

## SUPPLEMENTARY FIGURE AND TABLE



## Abbreviations

AIB1: amplified in breast cancer 1
HCC: hepatocellular carcinoma
VEGFR: vascular-endothelial growth factors receptor
PDGFR: platelet-derived growth factors receptor
PUMA: p53-upregulated modulator of apoptosis
Mcl-1: myeloid cell leukemia 1
GSK-3β: glycogen synthase kinase 3β
GADD45β: DNA damage-inducible gene 45β
c-IAP: cellular inhibitor of apoptosis proteins
MIF: macrophage migration inhibitory factor
eIF4E: eukaryotic initiation factors 4e
mTOR: mammalian target of rapamycin
p70S6K: p70 ribosomal S6 kinase
RP-S6: S6 ribosomal protein
4EBP1: eukaryotic initiation factor 4E binding protein 1
PARP: poly ADP-ribose polymerase
GCLC: catalytic subunit of glutamate cysteine ligase
GCLM: modifier subunit of glutamate cysteine ligase.
