# Supplementary material for: Downregulation of amplified in breast cancer 1 contributes to the anti-tumor effects of sorafenib on human hepatocellular carcinoma
Source: Oncotarget. 2016 Apr 18;7(20):29605–19. doi: 10.18632/oncotarget.8812 (PMC5045420; doi:10.18632/oncotarget.8812)
Supplement: Supplementary file 1 [file oncotarget-07-29605-s001.pdf]

SUPPLEMENTARY FIGURE AND TABLE

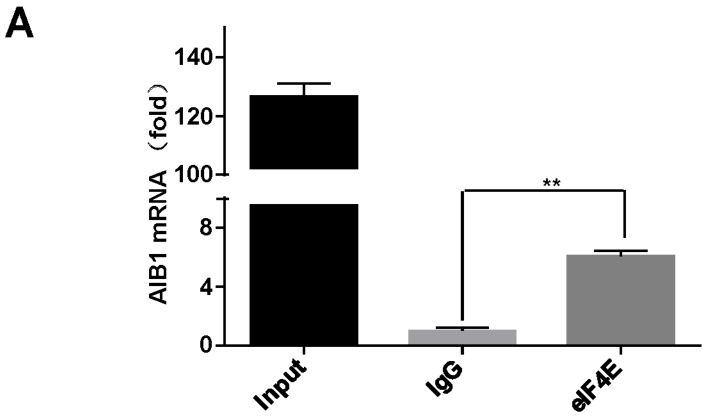

Supplementary Figure S1: AIB1 mRNA interacts with eIF4E. AIB1 mRNA was detected by real-time PCR after RNA immunoprecipitation using IgG or eIF4E antibody in HepG2 cells.

Supplementary Table S1: Primers for real-time PCR

Primer	Sequence
AIB1 forward	GGCTTTGTCTCAACCCACT
AIB1 reverse	CTCATGCCGCCTACCGAAC
GAPDH forward	CACTCCTCCACCTTTGACGC
GAPDH reverse	TGCTGTAGCCAAATTCGTTGT
MIF forward	CGGACAGGGTCTACATCAA
MIF reverse	CGGCTCTTAGGCGAAGGTG
Catalase forward	CTGTTGAAGATGCGGCGAGAC
Catalase reverse	TCCTGTGGCAATGGCGTTA
GCLC forward	CGGACAAGAATACACCATCTCA
GCLC reverse	ATACTGCAGGCTTGGAATGTC
GCLM forward	GGGAACCTGCTGAACTGG
GCLM reverse	CTGGGTTGATTTGGGAACTC
